# Usability and microbial validation study of an autonomous UV-C disinfection robot

**DOI:** 10.1016/j.infpip.2026.100582

**Published:** 2026-08-22

**Authors:** Thomas Roberto de Jager, Tijn Olivier Delzenne, Dennis Claessen

**Affiliations:** a Ryberg B.V., Delft, The Netherlands; b Institute of Biology Leiden, Leiden, The Netherlands

**Keywords:** UV-C disinfection, Microbiological validation study, Infection control, Autonomous decontamination

## Abstract

**Background:**

Hospital-acquired infections (HAIs) remain a major challenge in healthcare environments, leading to significant patient morbidity, mortality and increased healthcare costs. Automated ultraviolet C (UV-C) disinfection has been recognized as a potentially effective method to reduce microbial contamination on hard, non-porous, high-touch surfaces, minimizing the errors present in manual disinfection efforts.

**Aim:**

To evaluate the microbiological effectiveness of an autonomous moving UV-C disinfection robot (Omnia UV-C v1.0, Ryberg B.V., Delft, The Netherlands) through microbiological validation and usability assessment.

**Methods:**

The efficacy of disinfection was assessed by collecting surface swab samples from selected high-touch locations before and after robot disinfection, analysing microbial log reductions of indicator bacteria (*Escherichia coli*, *Bacillus subtilis* and *Pseudomonas putida*). Disinfection doses were measured with analogue dosimeters, and the robot's disinfection reports were analysed to correlate UV-C dose with microbial reduction. Static and dynamic disinfection runs were performed to evaluate the added value of automated movement during disinfection.

**Results:**

The robot achieved microbial log reductions exceeding 4 logs (99.99%) at various locations, meeting established disinfection criteria. Disinfection reports, dosimeter data, and microbial assays showed a strong correlation with dynamic disinfection, providing coverage advantages over static approaches. The disinfection doses measured were consistent with effective microbial decontamination.

**Discussion:**

The autonomous UV-C disinfection robot demonstrated effective microbial decontamination, highlighting its potential to aid in infection control in healthcare settings. Future work will include broader testing for various pathogens and shadowed areas to optimize disinfection coverage.

## Introduction

Hospital-acquired infections (HAIs) represent a significant challenge in healthcare settings, leading to increased mortality and healthcare costs [1]. These infections, caused by pathogens prevalent in the hospital environment, can lead to severe complications for patients, particularly those with a weakened immune system. Over 91,000 annual deaths in Europe alone can be attributed to HAIs [2]. Additionally, hospital settings prove to be the perfect breeding ground for multi-drug-resistant organisms (MDROs) [3,4], further complicating treatments. The persistence and spread of MDROs in healthcare environments highlight the limitations of traditional surface disinfection methods using pre-wetted disinfectant towelettes and spray bottles manually. Strict adherence to the hospital's disinfection protocols by means of continuous training of communication to staff is required to maintain compliance [5]. Detergent wipes currently used in healthcare facilities also repeatedly transfer pathogens to other surfaces [6]. In addition to this, their level of saturation varies due to storage [7], potentially affecting disinfection performance. Spray bottles, on the other hand, are likely to become contaminated over time [8]. Particularly high-touch surfaces, such as light switches, interfaces of medical devices, and rails of hospital beds, pose a risk for pathogen transmssion [9,10] and require frequent cleaning and disinfection. Operator-independent ultraviolet-C (UV-C) disinfection is not a replacement for manual disinfection practice as it cannot clean surfaces, but it can improve the overall disinfection performance if used as an additional safety layer [11], potentially decreasing the frequency of disinfection required to keep the microbial load within the hospital's acceptance criteria. As the system is automated, the frequency of quick or targeted disinfections can be increased if desired. Using traditional manual disinfection methods alone, an estimated 50% of high-touch surfaces in the operating room are missed by crews using manual disinfection approaches alone [12].

Traditional disinfection strategies involve manual disinfection using disinfectants, varying in effectiveness depending on the thoroughness of the disinfection process, their contact time [13], and the type of disinfectant used [14]. Solutions containing alcohol, chlorine, quaternary ammonium compounds, hydrogen peroxide or phenolic compounds are commonly used in this process.

In recent years, automated disinfection products have started to emerge as a promising solution to stream-line the hospital disinfection process. These devices provide a consistent and thorough approach to decontamination of non-porous high-touch surfaces, reducing reliance on manual disinfection alone. Among these innovations, UV radiation robots have gained particular attention for their effectiveness and efficiency. UV-C radiation is particularly effective in inactivating a broad spectrum of micro-organisms, including bacteria, viruses and spores, by inducing lethal mutations in their DNA or RNA [15,16]. This method of disinfection excels in its ability to reach and sanitize areas that may be overlooked or inadequately disinfected by human staff, with minimal downtime required. However, a recent study indicated that UV-C robots are not yet ready to be integrated in the cleaning and disinfection procedures, as the robots required many manual interventions, and the overall disinfections were not sufficient for day-to-day use [17].

This study evaluated the disinfection efficacy of an automated UV-C radiation robot on multiple bacterial targets. The micro-organisms used for this study were of microbial containment level 1 (ML-1; https://wetten.overheid.nl/BWBR0009653/2010-10-01#Bijlage5), as this was a restriction of the available laboratory. The list of ML-1 type organisms was cross-referenced against the available organisms at Leiden University, and excluded based on their relevance in hospital environments. Species were chosen as the closest representatives of prevalent pathogenic groups in hospital settings.

The effects of radiation time, intensity and distance were characterized to serve as a guideline for disinfection programmes. The primary objective of this research was to assess the feasibility of a current state-of-the-art UV-C disinfection robot for disinfecting hospital settings in accordance with international guidelines. The robot should not be used to target medical devices/instruments specifically, but for whole-room decontamination. It can be deployed any time of day or as a quick intervention between surgeries, where manual disinfection takes too much time. No people should be present in the same area as the device during the disinfection.

In addition to manual disinfection, hydrogen peroxide is often dispersed as a vapour for terminal decontamination of a room. While this technique is efficient at disinfection, it is laborious to incorporate, and often requires significant downtime for the area to be safe for use again. Both manual disinfection and fumigation are effective at disinfecting horizontal surfaces, so EN 17272:2020 uses vertical surfaces for evaluating disinfection efficacy. In contrast, most UV-C disinfection devices can disinfect vertical surfaces more easily due to the relative orientation of the light source. The BS 8628:2022 standard (derived from EN 17272) is therefore focused on horizontal surfaces instead. It should be investigated whether UV-C can potentially be deployed as an alternative to fumigation. The Omnia robot (Ryberg B.V., Delft, The Netherlands) was evaluated against the BS 8628 standard and achieved the results shown in Table I. However, the standard is focused entirely on static disinfection of a horizontal surface at a distance of 2 m and a height of 1–1.2 m. The aspect of having a dynamic moving robot is entirely negated in this standard. Moving the light source can reduce micro-shadow formation on surfaces, close the distance from source to target, and decrease the angle of incidence of the UV-C light on the surface. For these reasons, the standard is less applicable to dynamic moving disinfection robots, and the moving aspect should be investigated instead. This paper is doing exactly that, by comparing static versus dynamic moving UV-C disinfections.

**Table I:** Test results from static disinfection according to the BS 8628 standard

Micro-organism	Organism category	Disinfection type	Microbial log reduction
*Enterococcus hirae* ATCC 10541	Bactericidal	Static	5.0
*Staphylococcus aureus* ATCC 6538	Bactericidal	Static	7.1
*Acinetobacter baumanii* ATCC 19606	Bactericidal	Static	7.1
*Escherichia coli* ATCC 10536	Bactericidal	Static	4.0
*Candida albicans* ATCC 10231	Yeasticidal	Static	4.8

## Methods

This study aimed to determine the efficacy of the Omnia robot against *Escherichia coli*, *Bacillus subtilis* and *Pseudomonas putida* for a variety of robot settings. Static and dynamic disinfections were compared. Usability and set-up times were taken into account. The analogue UV-C dosimeters, robot disinfection reports and actual microbial log reductions were also compared. The acceptance criteria for bacteria was ≥5 log reduction of the microbial load in accordance with BS 8628.

### Room layout

The research laboratory shown in Figure 1a was used as the controlled environment for the disinfection experiments while emulating a hospital setting. Contrary to the BS 8628 standard, it is a furnished room, with objects forming natural areas of shadow and reflection. Laboratories are often used for testing with micro-organisms to mitigate the risk of contamination. A schematic layout of this room is shown in Figure 1c. The starting locations (S1, S2, S3 and S4) of the robot, the doors and control tablets (T1 and T2), and the microbial sample locations (A, B and C) are indicated on this figure. The disinfection area was set to be half of the room, with the remainder marked as a ‘no-go’ zone.

**Figure 1:**
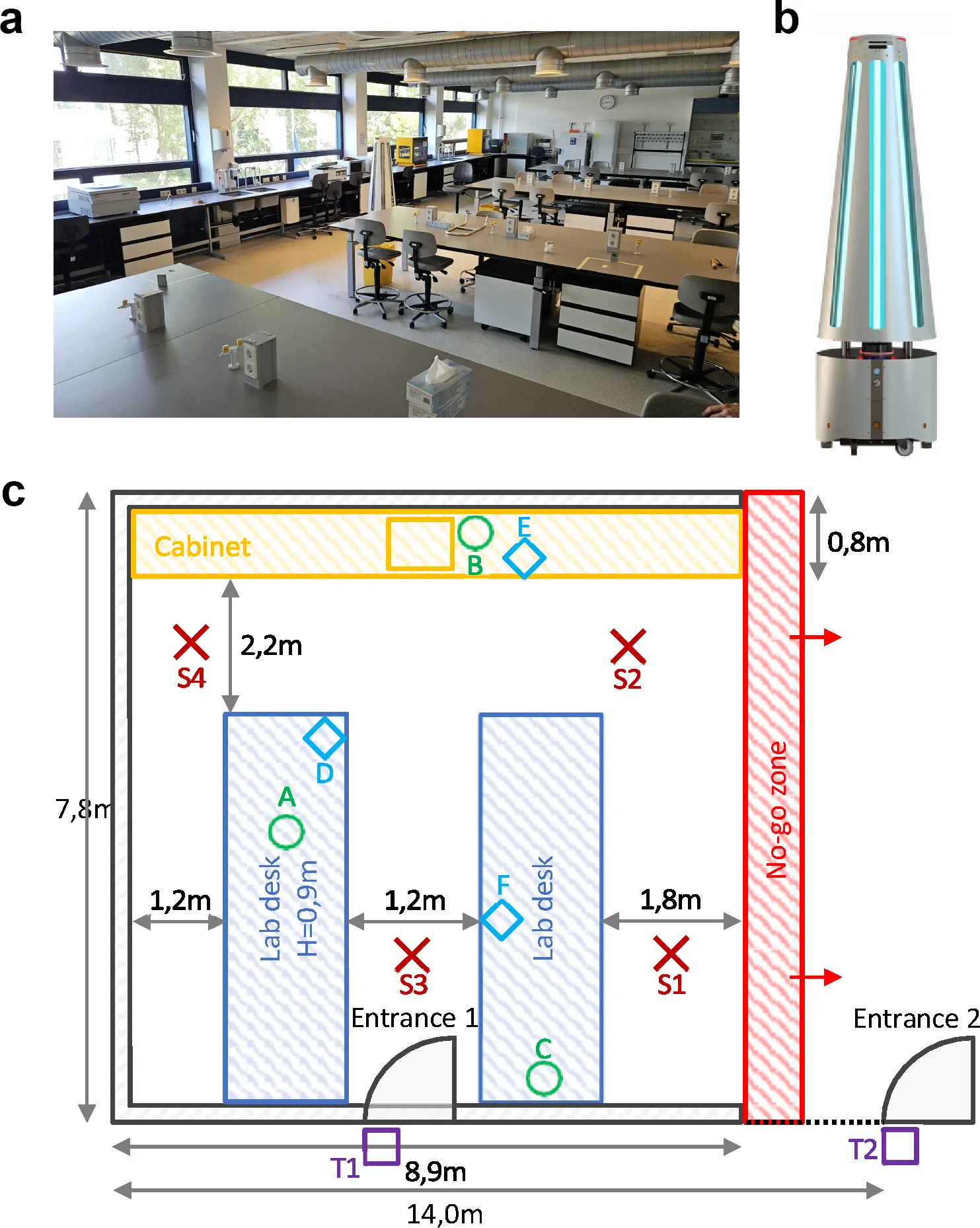
Layout of laboratory and robot used throughout the disinfection cycle. (a) Omnia robot in the test environment. (b) Omnia robot. (c) Schematic layout of the test environment. Squares indicate door guards, circles indicate microbial sample locations, diamonds indicate surface swab test locations, and crosses indicate start position of robot.

The sample locations were classified as either direct exposure (A, C, D and F) with >80% of disinfection time in direct sight of the robot and >80% of disinfection time within a 4-m radius of the robot, or indirect exposure (B and E) with 40∼80% of disinfection time in direct sight of the robot and 40∼80% of disinfection time within a 4-m radius of the robot. All sample locations were on a horizontal plane at a height of 0.9 m.

### Robot and settings used

The Omnia robot used in this study was of type OMNIA.UVC.GEN1.BSE1.21.01, with hardware version 1.00 beta 1 and software version 1.00 beta 4. The robot is shown in Figure 1b.

Four disinfection modes were defined, which can be set by the operator before each disinfection is started. The disinfection setting corresponds to a target dose in mJ/cm^2^. The target dosage values are estimates, and the resulting exposure values reflected in the disinfection report are based on algorithms created with empirical data. The quick disinfection setting targets 10 mJ/cm^2^, the normal setting targets 20 mJ/cm^2^, the deep setting targets 30 mJ/cm^2^, and a custom value could be set in the range of 40∼100 mJ/cm^2^.

The robot uses a full coverage path planner, making use of inward spirals, to cover the area to be disinfected at least once. Subsequently, the robot uses an internal feedforward estimate of the dosage to determine where it still needs to disinfect or which waypoints can be skipped, based on the target dosage set. The inflation radius of the robot is 15 cm, which is the closest the robot can get to an object. The maximum disinfection distance is a 3-m radius. The robot uses a non-holonomic differential drive to navigate. Due to the nature of the above methods, combined with the starting position and the objects encountered, the route of the robot is different for each disinfection.

### Disinfection programmes

Two types of disinfection were performed: static decontamination and automated dynamic decontamination. During static decontamination, the robot was placed manually in four predetermined locations within the room (S1, S2, S3 and S4, as defined in Figure 1c). At each location, a disinfection cycle was initiated and stopped manually after a set duration. During dynamic decontamination, the robot navigated the room autonomously, adjusting its path to achieve the target dosage. The robot's starting location is defined at S2, and it ceases operation once more than 85% of the autonomously generated waypoints were visited, and more than 90% received the target energy dosage. After each disinfection cycle, the set-up time, disinfection reports containing heat maps of disinfection with associated date and duration, and readings of the analogue dosimeters were collected. The analogue dosimeters used throughout the study were Intellego Technologies UVC254-DOTS (up to 100 mJ/cm^2^) for direct illumination and UVC254-QCC (up to 25 mJ/cm^2^) for shadowed areas. The dosimeters were placed next to each microbial plate, and were replaced after each disinfection.

### Bacterial strains and culturing

*E. coli* was chosen as a model for initial decontamination assessment due to its relevancy as a microbiological model organism and clinical Gram-negative pathogen. The strain to be tested was transformed with the pJUMP25-1A plasmid containing a kanamycin resistance gene (KanR) and superfolder green fluorescent protein (sfGFP), allowing for strict selection and visual confirmation of bacterial growth. *B. subtilis* was used as a safe working model for Gram-positive pathogens. This organism was used to study, amongst others, the pathogenic bacteria *Bacillus anthracis*, *Staphylococcus aureus* and *Listeria monocytogenes* due to its similar traits [18]. Lastly, *P. putida* was used as a model for *Pseudommas aeruginosa*, a common culprit in HAIs and one of the bacteria on the World Health Organization list of critical priority pathogens due to its high risk of multi-drug resistance. UV decontamination rates for these model organisms were cross-referenced with the pathogenic species they sought to replace to verify their validity as an accurate representation [19]. All bacteria used in this study were cultured on lysogeny broth (LB), which is known to support growth of all tested species. When required, 1% agar was added to create solid growth medium. For selection of *E. coli*, kanamycin was added to a final concentration of 50 μg/mL. *E. coli* and *B. subtilis* was cultured at 37 °C, while *P. putida* was grown at 30 °C. All liquid cultures were grown in an orbital shaker set to 200 RPM.

### Bacterial growth assessment

Colony-forming units (CFU) were used to assess bacterial growth under different conditions. For this, pre-cultures were inoculated and grown for 16 h, reaching the stationary growth phase. Optical density measurements were undertaken, and the culture was subsequently diluted down to OD_600nm_ = 0.1, after which it was grown to the exponential phase (OD_600nm_ = 0.4–0.8). After measuring the final density of the culture, serial dilutions were made in triplicate, ranging from 10^−1^ to 10^−7^. 10 microlitres of each sample was spotted on a square LB agar plate and allowed to dry for 20 min. These plates were subsequently exposed to UV decontamination programmes to quantify microbial survival. Additionally, during each decontamination test, one plate was not treated with UV. This would serve as the negative control. After treatment, plates were incubated for 24 h before the colonies were counted. The CFU values were determined using the final OD_600nm_ measurements of the liquid cultures and the colony counts. Log-reduction of each decontamination programme was calculated by comparing the CFU counts of the UV-treated plates with their respective untreated control plate.

#### Microbial characterization

16S rRNA gene sequencing was used to characterize bacterial species that survived UV decontamination. The 16S rRNA gene was amplified using universal primers 27F (5′-AGAGTTTGATCMTGGCTCAG-3′) and 1492R (5′-TACGGYTACCTTGTTACGACTT-3′). A small amount of the microbial colonies was taken and resuspended in the polymerase chain reaction (PCR) mix, containing Q5 polymerase, before a standard PCR protocol was executed. PCR conditions consisted of initial denaturation at 98°C for 2 min, followed by 30 cycles of denaturation at 98°C for 10 s, annealing at 63°C for 20 s, and extension at 72°C for 1 min, with final extension at 72°C for 5 min. Afterwards, the products were cleaned up using a NucleoSpin Gel and PCR Clean-up kit, whereafter samples were sent out for Sanger sequencing (Macrogen, The Netherlands). Pre-assembled sequences, obtained from Macrogen, were compared with the National Center for Biotechnology Information (NCBI) database using BLASTn to determine the closest known relatives.

## Results

### Set-up and disinfection characteristics

In order to assess the learning curve for using an automated UV decontamination device, the set-up time of the robot by a novice was measured throughout multiple experiments. Including booting the robot and tablets and waiting for 10 s before the lamps turn on, the time before starting disinfection ranged from 4 to 7 min in total, as reported in Table II. The operator used the robot once per week. The only intervention by the Ryberg staff after completing training was during the first disinfection. The operator had forgotten to release the emergency stop and therefore could not start disinfection. This was not indicated clearly on the control tablets.

**Table II:** Fluence values obtained from disinfection report and analogue dosimeters, with corresponding microbial log reductions and robot settings used for a variety of sample locations and micro-organisms

Organism	Robot setting		Duration (min)		UV-C fluence (mJcm^−2^)						Microbial log reduction [log]					
	Setting	Type	Set-up	Disinf.	Report			Dosimeter			Sample location			CFU remaining (avg.)		
					A	B	C	A	B	C	A	B	C	A	B	C
*Escherichia coli*		Static		13	4	2	4	20	5	10	>4	>3	>5	4 · 10^4^	8 · 10^4^	20
*Escherichia coli*		Static	5.7	28	12	6	12	25	15	20	>8^a^	>7	>7	0	23	7
*Escherichia coli*	Quick	Dynamic		13.21	>10	8	6	20	20	15	>7^a^	>7^a^	>7^a^	0	0	0
*Escherichia coli*	Normal	Dynamic	7.1	26.16	32	16	16	30	25	25	>6	>8^a^	>8	63	0	3
*Pseudomonas putida*	Normal	Dynamic	4.1	27.43	30	18	12	30	25	20	>5	>5	>5	18	650	28
*Bacillus subtilis*	Deep	Dynamic	4.9	40.42	50	25	20	65	30	35	>8	>7	>7	7	20	30

UV-C, ultraviolet-C; CFU, colony-forming units.

a All samples were fully sterilized.

The average disinfection time and disinfection area per disinfection mode were calculated from Table II and as follows. For a quick disinfection, the disinfection duration was, on average, 13.2 min for a surface area of 53.47 m^2^. For a normal disinfection, the mean ± SD duration was 26.96 ± 0.70 min for 53.66 m^2^. For a deep disinfection, the duration was 40.42 min with a surface area of 53.67 m^2^. The average disinfection time per square metre was 14.8 s/m^2^ for the quick disinfection setting, 30.1 s/m^2^ for the normal disinfection setting, and 45.2 s/m^2^ for the deep disinfection setting.

### Dynamic UV-C decontamination characterization

The estimated UV-C irradiance (energy levels in mJ/cm^2^) according to both the disinfection report of the robot and the actual irradiated UV-C energy are reported in the non-microbial results section of Table II. The values are reported for a variety of micro-organisms, robot settings and sample locations A, B and C in mJ/cm^2^. The starting position was S2 for each test, as shown in Figure 1c. An example of a generated robot disinfection report is shown in Figure 2. This indicates, on a two-dimensional map, where the robot went, how much UV-C irradiance the surfaces received, and the calculated log reduction values for the relevant bacteria. The tests were performed in 2024.

**Figure 2:**
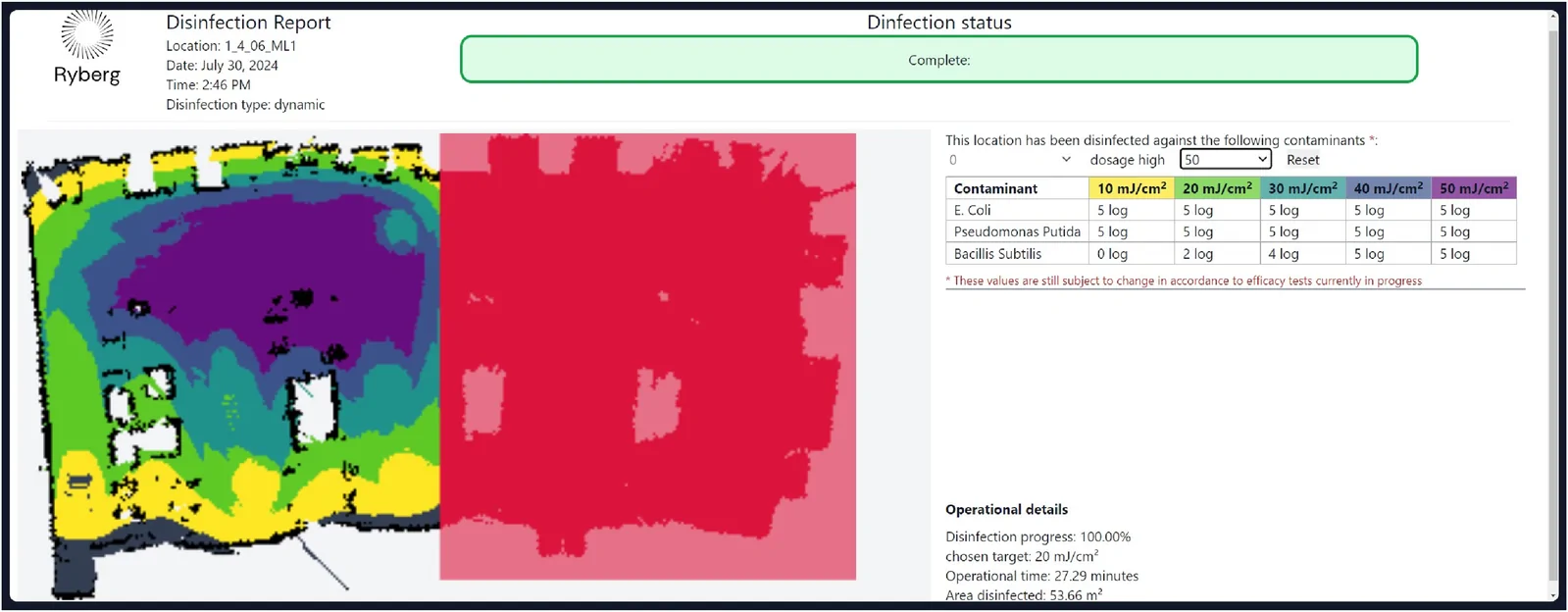
Example of a disinfection report generated by the Omnia robot after each disinfection.

For the disinfection in quick dynamic mode, the average irradiance was found to be >10, 8 and 6 mJ/cm^2^, respectively, according to the report and 20, 20 and 15 mJ/cm^2^, respectively, according to the dosimeters, resulting in a mean ± SD irradiance overall of 8.0 ± 2.0 mJ/cm^2^ according to the report and 18.3 ± 2.89 mJ/cm^2^ according to the dosimeters.

For a normal dynamic disinfection, the mean ± SD values were 30.7 ± 1.15, 18.0 ± 2.0 and 16.0 ± 4.0 mJ/cm^2^, respectively, according to the report and 30.0 ± 0, 23.3 ± 2.87 and 21.7 ± 2.87 mJ/cm^2^, respectively according to the dosimeters. The mean ± SD irradiance overall was 21.6 ± 7.26 mJ/cm^2^ according to the report and 24.4 ± 3.91 mJ/cm^2^ according to the dosimeters.

For a deep dynamic disinfection, the irradiance values were 50, 25 and 20 mJ/cm^2^, respectively, according to the report The dosimeters indicated 65, 30 and 35 mJ/cm^2^, respectively. The mean ± SD irradiance overall was 31.6 ± 16.1 mJ/cm^2^ according to the report and 43.3 ± 18.9 mJ/cm^2^ according to the dosimeters.

### Comparative decontamination of bacterial species

Several factors affect the rate of bacterial decontamination using UV light. Among these, the composition of the cell wall plays a significant role in the efficacy of UV-based bacterial decontamination. Gram-positive bacteria are generally more resistant due to their thicker peptidoglycan layer, which is located on the outer surface of the cell and blocks more light from reaching the DNA to induce germicidal mutations [20]. Furthermore, the formation of spores or the presence of light-blocking pigments can significantly impact the effectiveness of UV decontamination [21]. To test the influence of these factors on the decontamination rate, automated irradiation of multiple bacterial species was executed.

The total microbial log reduction values for *B*. *subtilis, P*. *putida* and *E*. *coli* are shown in Table II. The tests were performed at three different locations: A, B and C (see Figure 1c). Due to expected low decontamination rates, *B. subtillis* was decontaminated using the deep setting, while the other samples were irradiated using the normal setting. An example of the microbial plates used is shown in Figure 3a. *E. coli* was also disinfected using the quick setting, as it was expected to have a high decontamination rate. *E. coli* was found to be the easiest to decontaminate, reaching a 7–8 log reduction at all different locations using the normal decontamination setting. On the quick setting, a >7 log reduction for all locations was observed, with the samples at all locations fully sterilized. *P*. *putida* reached an overall log reduction of >5 when decontaminated using the normal setting. *B. subtilis* proved to be the most difficult to decontaminate, only reaching an average 7-log reduction when the deep decontamination setting was used. This setting multiplies the irradiance time over the normal setting used for the other bacterial species by a factor of approximately 1.5.

**Figure 3:**
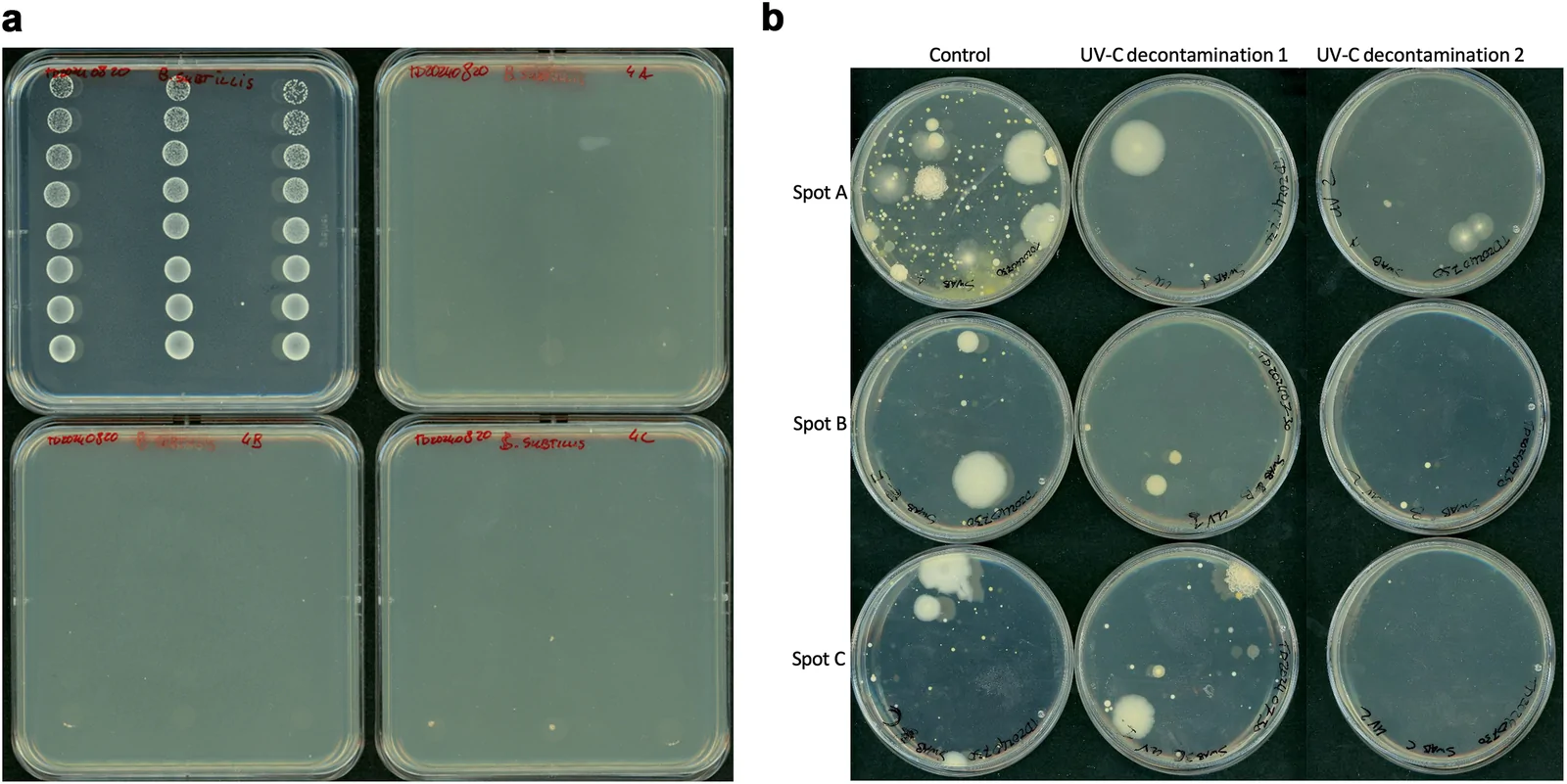
Examples of ultraviolet-C (UV-C) disinfection results using the Omnia robot. (a) Microbial plates of *Bacillus subtilis* after UV-C disinfection at the 30 mJ/cm^2^ dynamic disinfection robot setting. Top left plate is the control plate. (b) Surface culture swab result after incubation period.

### Static decontamination characterization

In addition to the dynamic disinfection mode, *E. coli* was also disinfected using the robot's static mode. This was done to assess the added value of autonomous disinfection over manual (static) UV decontamination strategies. Two tests were performed with a different duration, based on the disinfection duration observed with the quick and normal disinfection settings. The static test durations were chosen to be of equal length as dynamic quick and normal disinfection. The four starting positions were S1 to S4, indicated in Figure 1c. The results are presented in Table II. The quick static irradiance values according to the disinfection report were 4, 2 and 4 mJ/cm^2^, respectively, and the dosimeters indicated 20, 5 and 10 mJ/cm^2^, respectively. The mean ± SD irradiance overall was 3.33 ± 1.15 mJ/cm^2^ according to the report, and 11.67 ± 7.6 mJ/cm^2^ according to the dosimeters. This disinfection, with a total duration of 13 min, resulted in mean ± SD microbial decontamination values of 4.83 ± 0.42, 3.49 ± 0.04 and >5.78 ± 0 for sample locations A, B and C, respectively. During the normal static disinfection, the mean ± SD irradiance values were 12 ± 0, 6 ± 0 and 12 ± 0 mJ/cm^2^, respectively, for the report and 25 ± 0, 15 ± 0 and 20 ± 0 mJ/cm^2^, respectively for the dosimeters. The report indicated a mean ± SD value of 10 ± 3.1 mJ/cm^2^ and the dosimeters indicated a mean ± SD value of 17.5 ± 2.9 mJ/cm^2^. This longer static disinfection, with a duration of 28 min in total, resulted in a mean ± SD microbial log reduction of >8, 7.06 ± 0 and 7.70 ± 0 for locations A, B and C, respectively.

### Proof-of-concept surface decontamination

As a way to validate the previously obtained results, a proof-of-concept validation experiment was performed. During this protocol, surfaces naturally dirtied through the active and uncontrolled usage of the research laboratory were decontaminated and microbial load was investigated. For this, initial samples were taken by streaking a sterile swab over three pre-marked high-touch surface areas of 20 × 20 cm before applying the swab to an LB plate for growth. Automated UV decontamination was subsequently done in two phases, comprising one round of decontamination at the quick setting followed by a round at the normal setting. After each round, sampling of the microbial load in the areas was repeated. After all samples were obtained, plates were incubated at 30°C for 48 h. The resulting microbial growth can be seen in Figure 3b. Spot A, the corner of a table located in a high-traffic area of the laboratory, exhibited a relatively high amount of microbial growth. A distinct drop in growth can be seen between the sterilized surfaces and the first round of disinfection with a seemingly lessened effect by the subsequent round, indicating that the lowest setting of decontamination already eliminates the majority of the microbial growth.

After two rounds of radiation, colony formation persisted on multiple plates. To gain insights into these seemingly UV-resilient organisms, 16S rRNA sequencing was utilized to characterize the species capable of survival. For this, the 16S rRNA gene was amplified from three phenotypically distinct colonies and sent out for sequencing. Aligning the sequences against the NCBI database revealed the samples to have close similarity to *Arthrobacter* spp., *Lysinibacillus* spp. and *Staphylococcus* spp. Notably, all these species are Gram-positive.

## Discussion

Previous assessments of automated UV robots concluded that despite sufficient disinfection severity [22], the technology needed to be refined before devices could be deployed in a healthcare setting [17,23]. This study re-evaluates the usability and effectiveness of automated UV disinfection for healthcare settings, using a state-of-the-art automated robot. The results indicate that disinfection thresholds set by the US Environmental Protection Agency and the BS 8628:2022 standard (≥5 log reduction) can be reached while requiring minimal manual interventions during automated disinfection cycles, meeting the demands set previously [24].

Overall, the comparison of dosimeters with the microbial disinfection rates and the internal reports of the device showed that the actual log reductions equal or exceed the calculated reported log-reductions and measured values.

The disinfection values were obtained exclusively from smooth non-porous UV-C accessible surfaces. While most high-touch surfaces [[25], [26], [27]] meet this criterion, the need to identify shadowed areas and reflective surfaces [28] remains. Consequently, general guidelines or an assessment framework for optimal disinfection strategies should be developed.

Although standards such as EN 17272 and BS 8628 do not use a healthcare environment for validating efficacy, a limitation of this study is that a furnished laboratory environment was used to simulate a healthcare setting. A patient bed and a bathroom site, which are generally considered as challenging to disinfect, were not simulated.

Another limitation of this study is that the pathways inside the room were 1.2 m wide. In a hospital setting, pathways can be narrower. However, the robot can rotate around its longitudinal axis, and is designed and tested to operate in pathways of 0.8 m width. This is in line with the Americans with Disabilities Act standard for doorways and one lane corridors (width >815 mm).^1^

Similar to wheelchairs potentially contaminating other rooms as they are deployed in contaminated rooms [29], autonomous UV-C robots could suffer similar drawbacks not taken into account in this study. Additionally, power leads could be a potential source of cross-contamination.

It was found that the average disinfection time per surface area can be estimated by the following formula: *T* = 15 · *X* · *M*, where *T* is the disinfection time in s, *X* is the surface area in m^2^, and *M* is the disinfection mode setting of the robot. Quick disinfection is 1, normal disinfection is 2, and deep disinfection is 3.

In all dynamic *E. coli* disinfections, dosimeters confirmed that 10 mJ/cm^2^ was achieved with the resulting growth reduction exceeding 6-log. During static disinfection of *E. coli*, a mean ± SD log reduction of 3.49 ± 0.04 was found at dosimeter readings of 5 mJ/cm^2^, which falls in range of the expected 6.2 ± 3.7 mJ/cm^2^ found in the literature to be required for a 3-log reduction [19]. Dynamic disinfections of *P. putida* and *B. subtilis* met the 13 and 30 mJ/cm^2^ thresholds, respectively. While the fluence values required for a 5-log reduction were not measured for *B. subtilis*, microbial tests resulted in a reduction of at least 7-log. This heightened disinfection rate over previous studies could be due to differing rates in sporulation brought forward by differences in growth stage of the *B. subtilis* cultures, as formation of these more UV-resilient spores happens during high culture densities which this study did not test.

A comparison between static and dynamic disinfection protocols was performed to assess the added value of automated decontamination systems. The total duration of the static cycle, including set-up time between the different radiation locations, was matched to be the same as the total time of the dynamic cycle. The results from Table II show that for *E. coli*, a quick dynamic disinfection yielded higher fluence values from the disinfection report and dosimeters, resulting in higher decontamination rates. This could be attributed to less shadow formation as the robot was driving around objects, which was especially noticeable for sample location B. Additionally, during dynamic decontamination, the robot could come closer to the sample locations and the total exposure time was higher, as a result of not having to manually move and start the robot in between the static disinfections.

Comparing a dynamic disinfection at the normal setting with a comparable static disinfection, the results were similar, with sample location A showing a lower log reduction for dynamic mode, but sample locations B and C showing a higher log reduction. Sample location A was right next to two of the static disinfection starting locations. Location B, chosen for its partial shadow, was found to result in a higher log reduction in dynamic mode. These results indicate that static disinfection has a less even distribution of decontamination, causing localized peaks in overexposure while other areas are mostly untouched. This highlights the advantages of dynamic UV-C decontamination.

The Environmental Protection Agency has a 5-log reduction acceptance criteria for bacteria and 3-log reduction for viruses on products adding residual disinfection. Additionally, BS 8628:2022 specifies >5 log reduction for *E. coli*. However, traditional disinfectant wipes were tested to achieve efficacy of no more than 4-log reduction against bacteria, enveloped viruses, mycobacteria and yeast [30].

While the proof-of-concept experiment aimed to give a general overview of bacterial surface growth after decontamination cycles, inevitable selection will happen through the choice of growth duration, temperature and medium. This was minimized by allowing bacterial growth to happen over 3 days at a temperature of 30°C on LB agar, which allows for the growth of many heterotrophic bacteria and fungi. During testing, it was found that *Arthrobacter* spp., *Lysinibacillus* spp. and *Staphylococcus* spp. were able to grow after two rounds of quick disinfection. All these species are Gram-positive, which is of note because the thicker cell wall of these species is known to block UV-C at a higher rate than Gram-negative bacteria [31]. Additionally, *Arthrobacter* spp. are able to synthesize pigments and *Lysinibacillus* spp. can form spores, both of which heighten the resistance against UV-C even further [21].

It should also be noted that in part-shadowed areas, repair mechanisms of the micro-organisms, such as photoreactivation and dark repairs, could reverse part of the inactivation achieved [32].

While real-world environmental contamination is typically lower than the laboratory baselines used throughout this study, using a controlled contamination model is scientifically necessary to establish a standardized, experimental worst-case scenario compared with healthcare disinfection practices. In healthcare environments, routine cleaning and disinfection protocols aim to reduce surface contamination below known benchmarks. For example, Dancer's ‘safe surface concept’ states that high-touch surfaces are only safe when aerobic colony counts remain <5 CFU/cm^2^ and certain indicator organisms are completely absent [33,34]. However, testing a disinfection device against these already-low real-world baselines makes it statistically impossible to demonstrate the 4–5 log reductions required by regulatory standards. Therefore, the contamination model used throughout this study successfully bridges the gap between laboratory testing and field testing.

In conclusion, the effectiveness of an autonomous UV-C disinfection robot against *E*. *coli*, *P*. *putida* and *B*. *subtilis* was investigated. This study demonstrates that the robot is capable of achieving log reductions higher than currently used disinfection methods, and the disinfection reports generated provide an indication for actual microbial log reductions achieved. For regular disinfection speed settings, log reductions >7 for *E. coli*, >5 for *P. putida* and >7 for *B. subtilis* were observed. The device is capable of achieving the acceptance criteria for disinfection set by the US Environmental Protection Agency of ≥5 log reduction, and the BS 8628:2022 standard specifying ≥5 log reduction for *E. coli*.

This study has shown that UV-C disinfection with an autonomous robot is more effective than using a static device for the same disinfection duration and light intensity.

While previous research indicated that the technology readiness level of autonomous UV-C robots was not yet high enough to be used in daily cleaning and disinfection protocols, this was not observed during the course of this study. Practical insight into the robot set-up times and number of interventions needed is provided. Disinfection reports generated by the robot and the corresponding total disinfection times including disinfection severities are reported.

## CRediT authorship contribution statement

**T.R. de Jager:** Writing – review & editing, Writing – original draft, Visualization, Validation, Software, Resources, Project administration, Methodology, Investigation, Formal analysis, Data curation, Conceptualization. **T.O. Delzenne:** Writing – review & editing, Writing – original draft, Visualization, Validation, Software, Resources, Project administration, Methodology, Investigation, Formal analysis, Data curation, Conceptualization. **D. Claessen:** Writing – review & editing, Supervision, Resources, Funding acquisition, Conceptualization.

## Data availability statement

All data analysed in this study are included within this article, and raw data supporting the findings of this study are available from the corresponding author on request.

## Funding sources

This research did not receive any specific grant from funding agencies in the public, commercial or not-for-profit sectors. The authors received no financial support for the research, authorship and/or publication of this article.

## Conflict of interest statement

Ryberg B.V. is the manufacturer and sole provider of the Omnia UV-C disinfection robot used throughout this study. De Jager is an engineer at Ryberg. Leiden University did not receive compensation for performing this microbial validation study. Design, execution and quantification of the microbial decontamination experiments were performed independently by Delzenne, and were not influenced by the collaboration with Ryberg or De Jager. Neither the authors nor Leiden University have any financial interest or commercial relationship with Ryberg. Leiden University did not purchase, lease, receive or otherwise acquire a robot from Ryberg, and Ryberg had no involvement in the design, conduct, analysis, interpretation or reporting of this study.

## Footnotes

1 https://www.ada.gov/law-and-regs/design-standards/1991-design-standards/.
